# Supplementary material for: Label‐invariant models for the analysis of meta‐epidemiological data
Source: Stat Med. 2017 Sep 19;37(1):60–70. doi: 10.1002/sim.7491 (PMC5724693; doi:10.1002/sim.7491)
Supplement: Supplementary file 1 — Data S1. WinBUGS code for label‐invariant models [file SIM-37-60-s001.docx]

**Supporting Information: WinBUGS code for label-invariant models**

## Univariable model for the influence of a single study-level characteristic

**Data required:**

N_study=no. of studies

N=no. of studies x 2 arms

N_ma=no. of meta-analyses

*Study-level:*

treat=1 if treatment arm, 0 if control

r=no. of events

n= no. of participants

C1[i] = 1 if the *i*th study has the characteristic of interest, 0 otherwise

ma[i]<- meta-analysis index

s[i]<-study index

*Meta-analysis level:*

kappa_ok[m] = 1 if there are at least two studies with, and two studies without, the characteristic in the *m*th meta-analysis, 0 otherwise

**The WinBUGS model**

model{

for (i in 1:N) {

r[i] ~ dbin(p[i],n[i]) # likelihood (binary data r/n=no.of events/no. of participants)

logit(p[i]) <- alpha[s[i]] + treat[i]*(theta.minus[i]*+ beta[i] *C1[i])

# model (treat indicator of treatment group; C1 indicator of study characteristic)

beta[i]~dnorm(mean[ma[i]],p.k2[ma[i]])I(-10,10) # between study, within meta-analysis, variation in difference in effect associated with characteristic

theta.minus[i]~dnorm(d[ma[i]],p.d[ma[i]])I(-10,10) #RE for intervention effect within meta-analysis

rhat[i] <- p[i] * n[i] #calculate residual deviance

dev[i] <- 2 * (r[i] * (log(r[i])-log(rhat[i])) + (n[i]-r[i]) * (log(n[i]-r[i]) - log(n[i]-rhat[i])))

}

resdev <- sum(dev[])

for (j in 1:N_study) {alpha[j] ~ dnorm(0,.001)} # priors for study baseline effects - unrelated

for (m in 1:N_ma) {

mean[m] <- d[m] + b[m]

d[m] ~ dnorm(0,.001) # priors for true fixed (unrelated) intervention effects

b[m] ~ dnorm(b0,p.phi) # between meta-analysis variation in average difference in effect associated with characteristic

var_d[m]~dlnorm(mu,p.tau) # log-normal distribution for between-study variances

p.d[m] <- 1/var_d[m]

p.k2[m] <- equals(kappa_ok[m],1)/(var_d[m]*lambda)

+equals(kappa_ok[m],0)/(var_d[m]*cut(lambda))

}

#Prior for unknown parameters

b0 ~ dnorm(0,.001) # vague prior for overall average difference in effect associated with characteristic

lambda ~dlnorm(0,1) # vague prior for change in between-study variation associated with characteristic

p.phi1 ~ dgamma(.001,.001) # vague prior for between meta-analysis variation in average difference in effect associated with characteristic

phi <- pow(p.phi,-0.5)

p.phi <- p.phi1/(1-patom.phi)

patom.phi ~ dbeta(1,1)

mu~dnorm(0,0.001)

p.tau<-1/(sd.tau*sd.tau)

sd.tau~dunif(0,2)

log.tau2.new~dlnorm(mu,p.tau) # predictive distn for heterogeneity among studies without the characteristic

tau2.new<-exp(log.tau2.new)

# Parameters to monitor

q[1] <- b0

q[2] <- exp(b0)

q[3] <- lambda

q[4] <- phi

q[5]<- log.tau2.new

q[6]<-tau2.new

}

## Multivariable model for the influence of three study-level characteristics

**Data required:**

N_study=no. of studies

N=no. of studies x 2 arms

N_ma=no. of meta-analyses

*Study-level:*

treat=1 if treatment arm, 0 if control

r=no. of events

n= no. of participants

C1[i,j] = 1 if the *i*th study has the *j*th characteristic, 0 otherwise

ma[i]<- meta-analysis index

s[i]<-study index

*Meta-analysis level:*

kappa_ok[m,j] = 1 if there are at least two studies with, and two studies without, the *j*th characteristic in the *m*th meta-analysis, 0 otherwise

clambda[m,j] = 1 if there are 1, K-1 or K studies with or without the *j*th characteristic in the *m*th meta-analysis, 0 otherwise, where K is the no. of studies in the meta-analysis.

C0[m,j]=1 if there are no studies in the *m*th meta-analysis with the *j*th characteristic

**The WinBUGS model**

model {

for (i in 1:N) {

r[i] ~ dbin(p[i],n[i]) # likelihood (binary data r/n=no.of events/no. of participants)

logit(p[i]) <- alpha[s[i]] + theta[i]*treat[i]

theta[i]<-theta.minus[i]* (1-C1[i,1]) *(1-C1[i,2]) *(1-C1[i,3]) #effect in study without any of the characteristics

+theta.plus[i]* (1-(1-C1[i,1]) *(1-C1[i,2]) *(1-C1[i,3])) effect in studies with one or more characteristics

theta.plus[i] ~ dnorm(mean[i], p.k2[i])I(-10,10)

theta.minus[i]~dnorm(d[ma[i]],p.d[ma[i]])I(-10,10) #RE for treatment effects within meta-analysis

mean[i]<-d[ma[i]]+b[ma[i],1]*C1[i,1]+b[ma[i],2]*C1[i,2]+b[ma[i],3]*C1[i,3]

k2[i]<- ( (1-C1[i,1]) + # without characteristic 1

C1[i,1] * kappa_ok[ma[i],1] * lambda[1] + # with characteristic 1 and inform

C1[i,1] * clambda[ma[i],1] * cut(lambda[1]) + # with characteristic 1 but don't inform

C1[i,1] * C0[ma[i],1] *1) * # no studies have characteristic 1 in the MA

( (1-C1[i,2]) + # without characteristic 2

C1[i,2] * kappa_ok[ma[i],2] * lambda[2] + # with characteristic 2 and inform

C1[i,2] * clambda[ma[i],2] * cut(lambda[2]) + # with characteristic 2 but don't inform

C1[i,2] * C0[ma[i],2] *1) *# no studies have characteristic 2 in the MA

( (1-C1[i,3]) + # without characteristic 3

C1[i,3] * kappa_ok[ma[i],3] * lambda[3] + # with characteristic 3 and inform

C1[i,3] * clambda[ma[i],3] * cut(lambda[3]) + # with characteristic 3 but don't inform

C1[i,3] * C0[ma[i],3] *1)* # no studies have characteristic 1 in the MA

var_d[ma[i]]

p.k2[i]<-1/k2[i]

rhat[i] <- p[i] * n[i] #calculate residual deviance

dev[i] <- 2 * (r[i] * (log(r[i])-log(rhat[i])) + (n[i]-r[i]) * (log(n[i]-r[i]) - log(n[i]-rhat[i])))

}

resdev <- sum(dev[])

for (j in 1:N_study) {alpha[j] ~ dnorm(0,.01)} # priors for study baseline effects - unrelated

for(m in 1:N_ma){

d[m] ~ dnorm(0,0.01) # priors for true fixed (unrelated) intervention effects

for(j in 1:3){b[m,j] ~ dnorm(b0[j], p.phi[j])} # between meta-analysis variation in average difference in effect associated with characteristic

var_d[m]~dlnorm(mu,p.tau) # log-normal distribution for between-study variances

p.d[m] <- 1/var_d[m]

}

#Prior for unknown parameters

for(j in 1:3){

b0[j] ~dnorm(0,0.001) # vague prior for overall average difference in effect associated with characteristic

lambda[j] ~ dlnorm(0,0.1) # vague prior for change in between-study variation associated with characteristic *j*

p.phi1[j] ~ dgamma(0.001, 0.001)

phi[j] <- pow(p.phi[j],-0.5)

p.phi[j] <-p.phi1[j]/(1-patom.phi[j]) # vague prior for between meta-analysis variation in average difference in effect associated with characteristic

patom.phi[j] ~ dbeta(1,1)

exp.b0[j]<-exp(b0[j])

}

mu~dnorm(0,0.01)

p.tau<-1/(sd_tau*sd_tau)

sd_tau~dunif(0,2)

log.tau2.new~dlnorm(mu,p.tau) # predictive distn for heterogeneity among studies without the characteristics

tau2.new<-exp(log.tau2.new)

}
